# Cases of Poisoning by Arsenic

**Published:** 1849-09

**Authors:** 


### CHEMISTRY.

*Cases of Poisoning by Arsenic.*—At the Medical Society of London, Mr. Headland called attention to two cases that occurred in his neighbourhood. He was requested, on Sunday morning, to see a child; he found it pale, faint, had vomited largely and purged frequently, pain in the bowels—in fact, the child alarmingly ill. There was no satisfactory explanation of attack. Found the evacuations without colour, like thin gruel, here and there a bloody mark, then blood itself. The vomiting and purging continued until Wednesday, when the child was convalescent. On that day another child in the same family was taken ill, with similar symptoms. In this case there was no pain; the belly was slightly swollen. He was struck with the probability that it was the effects of mineral poison; an investigation took place into the culinary utensils, &c., without being able to account for the cause. The mother, however, stated that the child had been presented with a toy a few days before; it was in the shape of a white rabbit, and had been played with constantly the day previous. The child being timid, the nurse had some difficulty in causing it to place its hand on the toy and rub it, immediately after which the child put its hand into its mouth; during the first child's illness the second child had played with the toy. He examined the toy, and found it dusted over with a quantity of fine white powder, and felt convinced then that he had discovered the cause. He subjected the powder to all the tests for arsenic, which left no doubt that arsenic existed. He wondered at the quantity of powder, but found the greater part consisted of carbonate of lead. He referred to the common employment of arsenic to preserve skins and furs from moths, &c. He had questioned whether the toy was English or foreign; it was stated to be foreign, and was generally infested with moths; this white powder was used to destroy them. It was a hint to the profession to be alive to prevent such risks. In answer to questions, he stated it was clearly not a case of poisoning by lead, as no constipation, colic, or other symptom of poisoning by lead appeared, and the quantity of arsenic need not be large to cause such symptoms. The powder was so fine that it could be readily inhaled as dust. He exhibited a specimen of lead and arsenic derived from four or five grains of the powder.

Mr. Pilcher considered it a most important case, and referred to the different compositions for the protection of furs, and the danger that might arise from ladies' boas and muffs, &c., that they so frequently hold to their mouths, being preserved with a similar powder.

Mr. Stedman had seen the different experiments made by Mr. Headland, and considered that he had underrated the quantity of arsenic present. He had frequently seen shopkeepers dust the skins and toys with a white powder, and probably of the same composition.

Mr. Dendy expressed thanks to Mr. Headland for mentioning these very important cases; there was no doubt arsenic in some quantity existed, and was the cause; yet we see, occasionally, symptoms such as those described, caused not by anything of such a deleterious nature. We should certainly be on our guard, and consider that these cases merited every publicity, and should be generally known.—*Dublin Med. Press.*

